# Person-centered dementia care in home care services – highly recommended but still challenging to obtain: a qualitative interview study

**DOI:** 10.1186/s12913-021-06722-8

**Published:** 2021-07-22

**Authors:** Kari-Anne Hoel, Anne Marie Mork Rokstad, Ingvild Hjorth Feiring, Bjørn Lichtwarck, Geir Selbæk, Sverre Bergh

**Affiliations:** 1 The Research Centre for Age-related Functional Decline and Disease, Innlandet Hospital Trust, Brumunddal, Norway; 2 The Norwegian National Advisory Unit on Ageing and Health, Vestfold Hospital Trust, Tønsberg, Norway; 3 Faculty of Health and Social Sciences, Molde University College, Molde, Norway; 4 Faculty of Medicine, University of Oslo, Oslo, Norway; 5 Department of Geriatric Medicine, Oslo University Hospital, Oslo, Norway

**Keywords:** Dementia, Home care services, Home care, Nursing, Continuity of care, Person-centered care, People with dementia, Shared decision-making

## Abstract

**Background:**

Dementia is one of the main causes of disability and dependence in older people, and people with dementia need comprehensive healthcare services, preferably in their own homes. A well-organized home care service designed for people with dementia is necessary to meet their needs for health- and social care. Therefore, it is important to gain knowledge about how people with dementia experience the home care service and if the service responds to their wishes and needs. The aim of this study was to explore the experience of home care services among people with dementia, to understand the continuity in services, how the service was adapted to people with dementia, and how the patient experienced person-centered care and shared decision-making.

**Methods:**

We used a qualitative, exploratory design based on a phenomenological-hermeneutic approach and performed individual in-depth interviews with persons with dementia. A convenience sample of 12 persons with moderate to severe degrees of dementia from four Norwegian municipalities participated in the study. The interviews were conducted in February 2019.

**Results:**

The findings identified that the participants appreciated the possibility to stay safely in their own homes and mostly experienced good support from staff. They expressed various views and understanding of the service and experienced limited opportunities for user involvement and individualized, tailored service. The overall theme summarizing the findings was: “It is difficult for people with dementia to understand and influence home care services, but the services facilitate the possibility to stay at home and feel safe with support from staff.”

**Conclusion:**

The participants did not fully understand the organization of the care and support they received from the home care services, but they adapted to the service without asking for changes based on their needs or desires. Although person-centered care is recommended both nationally and internationally, the participants experienced little inclusion in defining the service they received, and it was perceived as unclear how they could participate in shared decision-making.

## Background

Dementia is one of the main causes of disability and dependence in older people, and people with dementia will need comprehensive healthcare services as the disease progresses [1]. To meet the increased need for healthcare services in the future, a common strategy in countries all over the world is that people with dementia should receive necessary care in their own homes and live at home for as long as possible [1, 2]. A well-organized home care service designed for people with dementia that considers the patient’s resources, preferences, and needs is necessary to tailor the service to the individual patient [3]. It is therefore important to gain knowledge about how people with dementia experience a home care service that is supposed to respond to their wishes and needs.

In Norway home care services are provided as a public statutory service. Patients who receive home care services represent a heterogeneous group due to differences in age, medical conditions, and need for help. Based on the patient’s needs for healthcare services, it is each municipality’s responsibility to adapt the service to the individual patient [4, 5]. Home care services in Norway are offered 24/7, and care and support related to personal needs are free of charge for the patient. In principle, there is no upper limit on how much help a patient can receive, but in practice, the amount of home care services depends on available resources in the municipality [6].

Previous research has investigated experiences related to the organization of home care services for people with dementia from both an organizational perspective and the perspectives of staff and the patient’s family [7–9]. However, there is limited research on the experience of people with dementia’ with the home care services. To facilitate home care services adapted to people with dementia, it is important to gain more knowledge about their experiences, preferences, and needs related to home care services.

Many older people wish to continue their way of living, regardless of their functional abilities and illness [10]. This might be important to maintain autonomy, and a feeling of belonging, even after the dementia disease affects the ability to perform ADL functions [11]. Autonomy as a human right is determined in the UN Convention on the Rights of Persons with Disabilities (UN-CRPD) using a broad definition of the term disability that includes people with dementia and states their right to make their own choices [12]. People with dementia depend on the staff’s communication skills and initiative to introduce shared decision-making [13]. The possibility for the person to be involved and heard is a central prerequisite for person-centered care, the strongly recommended approach in all dementia services as stated in the Norwegian national guidelines on dementia [5]. Person-centered care is described as a value base focusing on individualized, tailored care that maintains the perspectives of the person with dementia and facilitates positive psychosocial support [14]. Good communication can contribute to predictability and an individually tailored service adapted to people with dementia [15, 16], and thus influence how the patient experiences the quality of the service they receive [17].

Understanding how people with dementia experience the home care services is of major importance for providing a customized service that maintains the patients’ experience of predictability and supports people with dementia to live in their own homes as long as possible. The objective of the current study is to contribute with knowledge to existing research on how people with dementia experience home care services.

## Methods

### Aim

The aim of this study was to explore the experience of home care services among people with dementia, to understand the continuity in services, how the service is adapted to people with dementia, and the extent to which they experience person-centered care and shared decision-making.

### Design and settings

The study had an explorative qualitative design based on a phenomenological-hermeneutic approach. In-depth interviews of people with dementia receiving home care services were used to collect the data. The study recruited participants from four municipalities in the southeastern part of Norway.

### Participants

Twelve people with moderate to severe dementia participated in the study. The municipalities were selected based on previous research collaborations and were geographically dispersed and varied according to size and number of inhabitants. The participants were recruited by the home care services leader or the dementia coordinator in the municipality; eligible participants were persons with a diagnosis of dementia in their medical record or suspected cognitive decline equivalent to a mild, moderate og severe dementia identified by the staff.

The inclusion criteria were cognitive impairment identified by the staff based on an overall assessment of the patient, receiving a minimum of 15 min of home care services daily for the past 4 weeks, and being able to give informed consent to participation. Inclusion required communication skills that made it possible to share experiences of receiving home care services. The Clinical Dementia Rating Scale (CDR) was used by staff in home care services to assess the participants’ degree of cognitive impairment and dementia after inclusion. Participants who scored 0–0.5, corresponding to no dementia or slight memory loss would be excluded from the study. The participants’ characteristics are presented in Table 1.

**Table 1:** Participant characteristics (*n* = 12)

Gender	
→ Female	7
→ Male	5
Age	
→ 70–80 years	4
→ 81–90 years	6
→ ≥91 years	2
Marital status	
→ Lived alone/widowed	9
→ Lived with family	1
→ Married	2
Services	
→ Home care services ×1 pr. day	2
→ Home care services ×2 pr. day	5
→ Home care services >x2 pr. day	5
Day-care center in addition to home care services	9
Clinical Dementia Rating Scale (CDR; Hughes et al. 1983)	
→ 1 (mild cognitive impairment)	0
→ 2 (moderate cognitive impairment)	5
→ 3 (severe cognitive impairment)	7

### Data collection

The interviews followed a semi-structured interview guide with topics on the participants’ experience with the home care service (Table 2). It was used both open-and close-ended questions when it was considered that in some cases it was easier for the participants to answer close-ended questions. In addition, the participants were encouraged to describe what was important to them. Eleven interviews were conducted in the participant’s own home, while one interview was conducted at a day-care center. Three participants were accompanied by a family member during the interviews, but since the aim of the study was to investigate people with dementia’s experience, only information given by the participants was included in this study. The first author (KAH) conducted all interviews, which lasted from 16 to 50 min. The data were collected in February 2019.

**Table 2:** Interview guide

Main topics	Experiences with the home care services
1.	Tell me about the home care service you receive.
	What kind of service is it?
	How long have you been receiving home care services?
2.	Can you describe your general experience with the help you receive from home care services?
	How does it feel to receive help in your own home?
	Who provides the home care services?
	What impact do you have on the services provided?
3.	Do you have any thoughts about how your wishes and agreements on the help you receive from the home care services are followed up?
	What information do you receive?
	Are you able to participate in decisions?
	Is what is important to you been taken into consideration?
4.	If you had any wishes for a better home care service, what would it be?

### Preunderstanding

The first author is a nurse with several years of clinical experience in home care services. However, she was not working in any of the municipalities where the data collection took place. All authors have extensive experience in dementia care and experience related to research in home care services.

### Ethical considerations

The study was approved by Norwegian Center for Research Data (NSD no. 449466). All participants received information about the study from the staff in the home care services, as well as information about the opportunity to withdraw from the study at any time. The consent form was written in easy-to-understand language to increase patients’ ability to understand the information about the study. The staff in home care services evaluated ability to give consent to participate. Due to the forgetfulness associated with the disease the participants were contacted prior to the interviews to ask if an interview was still relevant to conduct. In this way the participants confirmed that they still wanted to participate in the interview. All participants gave their written informed consent and agreed to take part in the interview.

### Data analysis

The interviews were audio-taped and transcribed by a professional transcriptionist. The material was analyzed supported by NVivo.11 [18], using a qualitative content analysis following six steps inspired by Granheim and Lundman [19]. In the preparation phase, the first (KAH) and third (IHF) authors read through the transcribed material to get a full overview of the content. All transcribed interviews were included in the analysis. The six steps of analysis were: 1) meaning units related to the participant’s experience of receiving home care services were identified in the text; 2) the meaning units were condensed into descriptions close to the text; 3) the meaning units were extracted and labeled with codes; 4) based on a comparison of similarity and differences, the codes were grouped into subcategories; 5) subcategories sharing commonalities were abstracted into categories; and 6) the categories were summarized and reflected on to identity themes.

The first and third authors (KAH and IHF) made the initial analysis and discussed each step with the second author (AMR). All authors took part in discussions in the ongoing analysis and identification of the themes. Towards the end of the analysis, no new codes or categories appeared. It was therefore considered that the material had achieved saturation [20].

Examples from the analyzing process are shown in Table 3.

**Table 3:** Examples of the analysis of interviews

Meaning Unit	Condensed meaning unit	Code	Subcategory	Category
I do not remember when they (home care services) are coming. Because they just drop by … They have not been here regularly, just stopped by because they know me … to see how I feel. So, I have not thought about how long I have received home care services.	They have not been here regularly, just dropping by because they know me	They are not here on a regular basis, just stopping by because they know me	It is unclear why they are coming	**Security despite reduced comprehension of the services**
They (the home care service) are coming in the morning and in the evening. I appreciate that. I feel taken care of … And that’s very reassuring. I really do, and I feel safe. They are coming late at night and again in the morning. They are probably coming during the day as well, but I don’t think so, because they would tell me.	I appreciate that the home care service is coming, I feel taken care of, and that is reassuring	I feel taken care of, and that is reassuring	To be taken care of	

### Rigor

All authors ensured that data was properly collected and participated in discussions related to the analysis process. In addition, all authors actively participated in the writing and final reading of the manuscript. The consolidated criteria for reporting qualitative studies checklist (COREQ) was used to ensure the quality of all steps in the research process from planning the interviews, data collection, analysis, to reporting the results [21].

## Findings

Table 4 presents the findings revealed from the analysis, including the overall theme, categories, and subcategories. Four categories were identified from the analysis: 1) Feelings of security despite reduced comprehension of the services; 2) The quality of the relation with the staff seemed more important than continuity; 3) The desire to live in their own home seemed to increase their effort to adapt to the home care service; and 4) Experiences of limited opportunities for shared decision-making about needs and care.

**Table 4:** Findings presented as the overall theme, categories, and subcategories

**Theme**	**It is difficult for people with dementia to understand and influence home care services, but the services facilitate the possibility to stay at home and feel safe with support from staff.**			
**Categories**	**Feelings of security despite reduced comprehension of the services**	**The quality of the relation with the staff seemed more important than continuity**	**The desire to live in their own home seemed to increase their effort to adapt to the home care service**	**Experiences of limited opportunities for shared decision-making about needs and care**
**Subcategories**	To be taken care of To leave the responsibility to others Trust in the service It is not clear why they are coming	Relationship to staff The value of knowing the staff Busyness in the home care service The experience of a pleasant staff	A desire to live at home Accepting the service as it is Continuity of service less important	A desire for inclusion in decisions Decisions are made without the patient’s participation I think I can participate if I want to

### Feelings of security despite reduced comprehension of the services

It seemed that the participants did not fully understand the organization of the care and support they received from the home care services. The participants said they had not received any general information or explanation of the service or how the home care services worked for them. The way the home care service was organized and the kind of help they could expect appeared unclear. Additionally, the participants were unsure about how the service had been introduced, the content of the help they were offered, or when and how often they could assume the home care service to arrive.

*“I do not know their (the home care service’s) plan, what it is, whether they have scheduled days for each one, or whether it is only occasionally that they are coming. I do not know. But at least I want them to come a little more often.”* *(11)*

Some participants described the visits from the home care services as members of staff stopping by, only to chat. Furthermore, they conveyed that the staff checked to see if everything was fine, and whether they were in good health. Even though the participants were not sure what the home care service were supposed to do when they came, they appreciated these short visits. One participant said, *“They come by, talk and then they go again, cheerful and nice.”* *(4).*

The participants considered the home care service as easily accessible and, that the staff were nearby and available to be called upon if they needed help with anything. Additionally, the participants described it as a relief that the home care services were in contact with their general practitioner and took responsibility for securing their medication. They appreciated the staff carrying out practical tasks such as serving them breakfast. This security they experienced in receiving the necessary help was described as being taken care of and being safe.

*“They are coming in the morning and in the evening, I appreciate that. I feel taken care of ... And that's very reassuring.” (08)*

### The quality of the relationship with the staff seemed more important than continuity

The participants described the staff as friendly and helpful; overall, they were satisfied with the care and support they received from the home care services. Still, some differences emerged in their considerations of the members of staff visiting them. They conveyed how they appreciated some staff more than others, as one participant said, “*I like some of the staff better than others”* *(11)*. Some of the staff were particularly competent care workers and facilitated their wishes in ways they more preferred; “Some are particularly good and precise in their work, and they serve breakfast they know I like” *(12)*.

The participants expressed the relationship with the staff as valuable and familiar: “*We become a family” (06).* Most of them expressed that it was nice to get to know the staff, and they seemed to share a responsibility to contribute to a trusting relationship in the conversations with staff. It could be challenging for them to establish trust and a relationship with the member of staff, as they regularly had to collaborate with different staff members when the continuity in staff was experienced as low. As one participant expressed, *“If there are new ones (staff), you have to break the ice. This is not necessary if we know each other” (10)*. According to the participants, it seemed easier to get to know the staff and have something to talk about if the same individuals came on a regular basis. Even though the participants experienced having many different staff members to relate to, it was not considered a major problem.

*“There are different people who come by, several times a day, I do not know everyone, but it is not so important.” (06)*

*“I like talking to strangers, so for me it does not matter that there are new ones (staff from the home care services).” (08)*

### The desire to live in their own home seemed to increase their effort to adapt to the home care service

The participants showed an accepting attitude in which they largely appreciated the care and support from the home care services as it was without suggesting changes. The participants expressed gratitude for being able to continue living in their own home and receive the help they needed there.

*“I'm very grateful, I am, because you never know … And in this case, in my situation, you cannot predict anything. You must take the day as it comes and live through it until the next day. But I am very happy that the home care service is coming.”* *(11)*

Through statements related to the acceptance of receiving help, participants described their gratitude for being able to live at home. The experienced value of being taken care of seemed to be more important than the need for home care staff to arrive on time or being familiar with the staff, even some occasionally could experience the service as unpredictable, The participants expressed a desire to adapt to the home care services and not be a bother to the staff.

*“I am grateful that I am being monitored in a way and that I am taken into account in every possible way. At least it's good to know that you can be safe and that you will be taken care of.” (08)*

The participants seemed to share an understanding that the staff had many patients in need of help, and hence, their time to help each of them could be limited. The participants who experienced the staff to be in a hurry when they came to visit tried to not be a nuisance to the staff by asking for extra time and help.

*“I'm waiting; it's not just me, you know, so it might be a little late at night before they are available. Then they are tired and are about to go home. I understand that.”* *(11)*

### Experiences of limited opportunities for shared decision-making about needs and care

The participants’ experiences in influencing the service provided for them seemed to vary. Some described a feeling of receiving care and support without asking for the help themselves. It was only to a small extent that they described the help they received from the home care services as tailored to their individual needs or preferences. Furthermore, the experience of being involved and asked about their need for help was limited. The care and support they received was described as mainly predetermined and based on the service’s incorporated routines and schedules and determined independent of the participants’ wishes and needs.

*“They usually come and have their routines, some to wash and all that stuff. Beyond that, I do not know. These ladies who come and want to talk, so I cannot exactly say anything about it … but I manage most things on my own.” (09)*

*“There was probably a plan that someone should come. Yes, it is like that in the village; they have some control over the old people, but that's fine.” (13)*

The participants were not sure about how much they could participate in decisions related to the service they were offered, but they assumed they would be able to decide if something was important for them. One participant explained, “*If there’s something that really matters, I’ll probably get to decide something, at least I think so.” (13)*

Some participants had a desire to be involved in the care and support offered to them. However, they were not sure how they could get involved and how much they could decide upon. Several participants expressed a desire for more frequent visits, more help related to housecleaning or assistance in getting outdoors. Even though participants experienced that the service was largely decided in advance, the staff encouraged them to express their wishes and needs.

*“I think they have mostly decided in advance the service I get. Maybe I can be involved in making decisions, but I have not tried.” (06)*

*“They encourage me to call them if I need any help, but I do not need it yet.” (07)*

### Overall theme

The final interpretation of the findings identified that the participants appreciated the possibility to stay safely in their own homes, and most experienced good support from staff with few unmet needs. However, they expressed various views and understandings of the service and had limited opportunities for involvement and individualized tailored services. The overall theme identified in summarizing the findings was: “It is difficult for people with dementia to understand and influence home care services, but the services facilitate the possibility to stay at home and feel safe with support from staff.”

## Discussion

This study aimed to explore the experience of home care services among people with dementia, to understand the continuity in services, how the service is adapted to their needs, and the extent to which they experience person-centered care and shared decision-making.

Our findings indicate that even though national authorities strongly recommend person-centered dementia care, there might be challenges in achieving this for patients with dementia receiving home care services. The findings reveal that people with cognitive disabilities can have difficulty in understanding the organization of the service, but the desire to live at home might increase the patients’ effort to adapt to the service rather than the home care service tailoring the care and support to the individual patient. Furthermore, the participants express low expectations of participation in planning and decision-making. In the following, we will discuss the main findings in relation to previous research and potential clinical and organizational implications.

### Home care services can be difficult to understand but still create security and good relationships

Based on international and national recommended standards for home care services offered to people with dementia, person-centered care and support based on the patient’s wishes and needs should be provided. Nevertheless, our study shows that the home care service appears to be difficult for patients to understand. They experience a high degree of uncertainty about how the service is organized, when and why the staff is coming, and what type of care and support they will receive. It turns out that information given to people with dementia can often be deficient and only slightly adapted to the challenges that accompany the disease, such as their difficulties in understanding and remembering information [22, 23]. This can present challenges in providing information about the service and tailoring the care and support to their wishes. Previous research has shown that a trusting relationship and continuity of staff may have a positive effect on the patient’s experience of the service [22, 24]. As the results in this study indicate, there is a heterogeneity in staff who provide the service in terms of knowledge and personality; some staff were described as particularly competent care workers who did a better job than others. Therefore, ensuring that the staff has sufficient knowledge about dementia may be important for customized service [25, 26]. This can contribute to good quality of care [27, 28] and better communication and cooperation between people with dementia and staff [7].

Even though the participants did not fully understand the organization of the care and support they received from the home care service, they were generally satisfied with the service and cared for. A trusting relationship between people with dementia and staff can be important in terms of providing person-centered care [29, 30] and might also increase their feeling of security [17]. Participants experienced a trusting relationship as more important than the same staff visiting them regularly. Nevertheless, previous research shows that low continuity in staff visiting the patient can make it difficult to establish good relationships with people with dementia [22, 31]. We can therefore assume that continuity of care and trusting relationships might be factors that influence each other and cannot be seen as independent.

### Low expectations and adapting to the home care service received

The findings in the current study might indicate that some people with dementia accept the care and support they receive as passive recipients with an overall desire to be satisfied with the service. Previous research has identified that care and support from home care services are described as standardized and based on routines that focus on practical tasks, and this can result in limited individualization to the patient’s needs [24, 27, 28]. Furthermore, the communication between staff and the person with dementia during visits can become task-oriented [15, 25]. Traditionally in Norway, home care services for people with dementia have been allocated based on observed needs. This may have contributed to the services provided being task-oriented more than based on the patient’s expressed wishes and needs. With limited resources for each patient, it can be challenging to arrange task-oriented service for each patient in accordance with the recommendation for personalized service for people with dementia [26], which can contrast with the recommendation for person-centered care [2, 3, 7, 30, 32]. Likewise, challenges associated with high workload in the service [33–36] can affect the staff’s ability to provide person-centered care for people with dementia.

Despite these challenges, participants expressed gratitude for being able to live at home, where they felt a sense of belonging and could be in familiar surroundings. To a large extent, they accepted the help they received from the home care service and did not want to be a nuisance to the staff. This is consistent with previous research that finds that people with dementia largely accept the service as it is without making demands regarding quality or adaptation [31]. However, this does not mean that being included in decisions about wishes and needs is less important for people with dementia. On the other hand, the desire to not bother the staff by asking for help can be reinforced by the experience of a busy home care service [22]. The results of this study may therefore have implications for practice through an increased awareness that care and support for people with dementia may be tailored to the service more than the patient, as people with dementia seem to adapt to the home care service without expressing their own needs. Nevertheless, the participants did not consider the quality of the service to be poor, which may indicate that the service is already adapted to the individual patient and they therefore feel satisfied with the service.

### The goal of shared decision-making is difficult to achieve

Even though it is recommended that people with dementia be involved in decision-making concerning their own health and the service provided [4, 7], this seems to be a challenge. The participants in the present study were unsure of their opportunity to influence the service provided. Some participants wanted to participate more but experienced that the care and support was determined in advance. Others said they were happy with the help they received and did not want to be included in decision-making. This coincides with previous studies revealing that people who receive home care service largely accept decisions made by others [37, 38]. For people with dementia to participate in shared decision-making, there is a need for sufficient knowledge about the service received and customized information about care and support [7]. This presupposes staff awareness of the challenges people with dementia have with understanding information and the need for assistance [37, 39]. Since people with dementia may have problems remembering the information provided, they should be offered numerous opportunities to talk about their wishes and needs, and the information should be provided in a written form [7, 37]. However, supported decision making might be useful to ensure that people with dementia are involved in the home care service they receive and thus be in the center of decision making [40].

Both in Norway and internationally, patients’ involvement in organizing their own services and striving for care and support to be tailored to patients’ wishes and needs are considered essential [4, 41]. Based on the participants’ experiences, this may indicate that the lack of knowledge about the care and support they receive, as well as an experience that the service is predetermined, challenge the opportunity to participate in decisions. Participants perceived the home care service as accessible, where the staff repeatedly encouraged them to state their own wishes. This may indicate that the home care service wants to facilitate participation, but the possibility is limited to the care and support already offered [31]. Nevertheless, we cannot exclude that the staff asked the participants about any wishes or needs for help during the scheduled visits without this resulting in an experience of being involved in their own service. The participants expressed it was difficult to receive assistance with housecleaning or going outdoors. Thus, it seems that financial and administrative resources in the service largely affect how the staff can tailor the service for the individual patient, which perhaps precludes person-centered care based on the patient’s wishes and needs.

The participants had moderate to severe degrees of dementia, but the experiences that emerged in this study clearly show the importance of listening to the experiences of people with dementia in receiving home care services. The study indicates that people with dementia only to a small extent are included in shared decision-making. Thus, they become participants without power, in a home care service that is largely determined in advance and limited by available resources. How to systematically facilitate person-centered care for people with dementia in home care services should receive increased attention in the future.

### Limitations and strengths of the study

All participants had a moderate to severe degree of dementia, which made it challenging to ask open-ended questions where they could speak freely. Instead, it was necessary to ask several closed-ended questions with necessary follow-up questions due to yes or no answer which may have influenced the participants’ answers. Further, we cannot exclude that participants did not remember what services they received, and this may have affected the results of the study.

The material was analyzed and interpreted by two of the authors together, with a third author as a discussion partner for each step in the analysis process. Nevertheless, the first author’s experience as a nurse in the home care service and work experience with people with dementia may have contributed to a positive view in the analysis and interpretation of data, as well as how the results are presented. However, the first author’s previous experience with people with dementia may have contributed to the interviews providing richer data material.

Three participants were accompanied by a family member during the interviews. Even though only information provided by the participants is included in this study, the presence of a family member may have influenced the information the participants shared in the interviews. None of the authors knew the participants in advance. The staff who included participants in the study may have selected participants who were more satisfied with the service than others, and thus influenced the outcome of the study. However, a strength of the study is that CDR was used as an inclusion criterion to support the discretionary assessment of the degree of dementia performed by the staff.

One strength of the study is inclusion of people with moderate to severe dementia which might increase the transferability of the findings. In addition, the participants are from several municipalities, geographically dispersed and with variation according to size and number of inhabitants. This also increases the transferability of the findings.

## Conclusion

For people with dementia to experience a personalized and tailored home care service, we consider that the organization of the home care service can be difficult to understand. In addition, the schedule of the day and limited time for each patient can challenge the staff’s ability to provide person-centered care and support. Although person-centered care has been a recommendation in care and support for people with dementia, it can be challenging to achieve in home care services. The results of this study indicate that people with dementia have a desire to live in their own home and therefore adapt to the service without asking for changes based on their own wishes or needs. This may indicate a greater responsibility for the staff in organizing the care to facilitate service to the individual patient. The findings show that people with dementia experience little shared decision-making in the service they receive, even though this is an essential part of person-centered care. Altogether, the findings from the current study challenge the responsibility of staff’ and care providers to include patients with dementia both in offering tailored information and facilitating user involvement by actively encouraging shared decision-making. Future research should shed light on how people with dementia should be supported to be able to understand the service they receive, as well as how to facilitate implementation of person-centered care in the service.

## Data Availability

The dataset used and/or analysed during the current study available from the corresponding author on reasonable request.

## Abbreviation

CDR: Clinical Dementia Rating Scale
