# Correction: Person-centered dementia care in home care services – highly recommended but still challenging to obtain: a qualitative interview study

**DOI:** 10.1186/s12913-022-08312-8

**Published:** 2022-07-22

**Authors:** Kari-Anne Hoel, Anne Marie Mork Rokstad, Ingvild Hjorth Feiring, Bjørn Lichtwarck, Geir Selbæk, Sverre Bergh

**Affiliations:** 1 The Research Centre for Age-related Functional Decline and Disease, Innlandet Hospital Trust, Brumunddal, Norway; 2 Faculty of Medicine, University of Oslo, Oslo, Norway; 3 The Norwegian National Advisory Unit on Ageing and Health, Vestfold Hospital Trust, Tønsberg, Norway; 4 Faculty of Health and Social Sciences, Molde University College, Molde, Norway; 5 Department of Geriatric Medicine, Oslo University Hospital, Oslo, Norway

**Correction: BMC Health Serv Res 21, 723 (2021)**

**https://doi.org/10.1186/s12913-021-06722-8**

Following publication of the original article [1], the authors identified an error in the affiliations assignment: the first author, Kari-Anne Hoel, should have had the Faculty of Medicine, University of Oslo (affiliation 2), as affiliation in addition to The Research Centre for Age-related Functional Decline and Disease, Innlandet Hospital Trust (affiliation 1).

This error is corrected in the author list of this Correction article and the original article [1] has been updated.
